# Case Study of Resilient Baton Rouge: Applying Depression Collaborative Care and Community Planning to Disaster Recovery

**DOI:** 10.3390/ijerph15061208

**Published:** 2018-06-08

**Authors:** Robin Keegan, Leslie T. Grover, David Patron, Olivia K. Sugarman, Krystal Griffith, Suzy Sonnier, Benjamin F. Springgate, Lauren Crapanzano Jumonville, Sarah Gardner, Willie Massey, Jeanne Miranda, Bowen Chung, Kenneth B. Wells, Stephen Phillippi, Ed Trapido, Alexa Ramirez, Diana Meyers, Catherine Haywood, Craig Landry, Ashley Wennerstrom

**Affiliations:** 1 Resilient Baton Rouge, 100 North Street, Suite 900, Baton Rouge, LA 70802, USA; williemfarve@gmail.com; 2 Semel Institute for Neuroscience and Human Behavior, UCLA David Geffin School of Medicine, 10833 Le Conte Ave, Los Angeles, CA 90095, USA; Dpatron@mednet.ucla.edu (D.P.); KGriffith@mednet.ucla.edu (K.G.); jmmiranda@mednet.ucla.edu (J.M.); BChung@mednet.ucla.edu (B.C.); 3 School of Medicine, Section of Community and Population Medicine, Louisiana State University Health Sciences Center, 433 Bolivar St, New Orleans, LA 70112, USA; okacsi@lsuhcs.edu (O.K.S.); bspri2@lsuhsc.edu (B.F.S.); sphill2@lsuhsc.edu (S.P.); etrapi@lsuhsc.edu (E.T.); arami2@lsuhsc.edu (A.R.); 4 Executive Director, Baton Rouge Health District; suzy.sonnier@brhealthdistrict.com; 5 Baton Rouge Area Foundation, 100 North Street, Suite 900, Baton Rouge, LA 70802, USA; ljumonville@braf.org (L.C.J.); sgardner@braf.org (S.G.); 6 St. Anna’s Episcopal Church, 1313 Esplanade Ave, New Orleans, LA 70116, USA; diana@stannanola.org; 7 Louisiana Community Health Outreach Network, 1226 N. Broad, New Orleans, LA 70119, USA; chaywoo@tulane.edu; 8 UCLA Center for Health Services and Society, Los Angeles, CA 90095, USA; cmbl@prodigy.net; 9 Department of Medicine, Tulane University School of Medicine, 1430 Tulane Ave. SL-16 New Orleans, LA 70112, USA

**Keywords:** disaster, community resilience, behavioral health, collaborative care, community health workers, cognitive behavioral therapy, depression

## Abstract

Background: Addressing behavioral health impacts of major disasters is a priority of increasing national attention, but there are limited examples of implementation strategies to guide new disaster responses. We provide a case study of an effort being applied in response to the 2016 Great Flood in Baton Rouge. Methods: Resilient Baton Rouge was designed to support recovery after major flooding by building local capacity to implement an expanded model of depression collaborative care for adults, coupled with identifying and responding to local priorities and assets for recovery. For a descriptive, initial evaluation, we coupled analysis of documents and process notes with descriptive surveys of participants in initial training and orientation, including preliminary comparisons among licensed and non-licensed participants to identify training priorities. Results: We expanded local behavioral health service delivery capacity through subgrants to four agencies, provision of training tailored to licensed and non-licensed providers and development of advisory councils and partnerships with grassroots and government agencies. We also undertook initial efforts to enhance national collaboration around post-disaster resilience. Conclusion: Our partnered processes and lessons learned may be applicable to other communities that aim to promote resilience, as well as planning for and responding to post-disaster behavioral health needs.

## 1. Background

There is increasing attention internationally toward the mental health consequences of disasters, which are often thought to be transient, but reported to persist in high prevalence in major events such as the post-Katrina floods or the Exxon Valdez oil spill [1,2]. Aspects of disasters associated with increased psychological distress and general health decline include: (1) physical injury and trauma; (2) displacement and damage to housing; (3) damage to property; (4) loss of or separation from loved ones; (5) loss of employment and other roles; (6) disruption in social networks and supports; (7) re-distribution of services; and (8) exposure to hazards [3]. However, a recent NIEHS report on climate and health noted that psychological consequences of disaster remain relatively under-studied as a public health outcome [4].

A key concept in promoting well-being is resilience, which refers to “a dynamic process encompassing positive adaptation within the context of significant adversity” [5]. Community resilience refers to capacities of communities or social structures to withstand challenges such as disasters across individuals in a community [6], and enhancing community resilience is a national goal [7]. Key dimensions of community resilience include social inclusion and connectedness [8], involvement of community residents and stakeholders in participatory planning and development to enhance community social capital [8] and assets [9] and promoting collaboration of informal and formal support systems. In this article, we describe the origin and initial development of the Resilient Baton Rouge program, which is designed to promote psychological well-being and reduce the burden of the mental health consequences of the Great Flood of 2016 in the Baton Rouge region. The goal is to provide a case study that illustrates how in the context of a disaster, community participatory planning can be combined with evidence-based mental health interventions adapted to the local context, building on a history of collaboration across Southern Louisiana and Los Angeles in resiliency-oriented mental health disaster recovery resources.

Baton Rouge context: The Great Flood of 2016 brought over seven trillion gallons of rain to Baton Rouge, Louisiana, in just 36 hours, causing over $20 billion in property damage and damaging or destroying nearly one third of homes in the region [10]. Approximately, 6000 businesses were flooded, and more than 278,000 of residents were unable to return to work [10]. High rates of symptoms of depression, anxiety and/or post-traumatic stress disorder (PTSD) persist in the months and even years after disasters, creating a need to expand services and outreach for mental health [1]. Even prior to the Great Flood, Baton Rouge was limited in its capacity to deliver behavioral health services, in part due to the 2013 closure of the public hospital, Earl K. Long, which had served as the primary source for care for people living in poverty or without health insurance [11]. East and West Baton Rouge Parishes were designated mental health professional shortage areas for low-income populations [12]. An evaluation of a temporary state-run crisis counseling program that began in the immediate aftermath of the disaster also indicated a need for additional, longer term behavioral health services [13].

History of recovery collaboration: The approach described in this case study of Resilient Baton Rouge builds on a 13-year history of collaboration in addressing adult depression and promoting mental health recovery post-disaster from a resilience perspective. In particular, in 2005, major levee failures associated with Hurricanes Katrina and Rita caused catastrophic flooding and largely decimated the local healthcare infrastructure in New Orleans. During this period, community and academic partners in Los Angeles had been developing a community-partnered, participatory approach to engaging under-resourced communities of color in addressing depression [14,15]. With technical support from collaborators from this Los Angeles team, as well as the AIMS Center at University of Washington, the New Orleans community and academic leaders implemented a multi-year project that enhanced local capacity to deliver depression care services that used resources from IMPACT [16], WeCare [17] and Partners in Care [18] adapted to New Orleans communities and the post-disaster context [19,20,21,22,23]. Major adaptations were mental health outreach through community health workers (CHWs) and other non-clinically trained community members such as neighborhood association staff. Roles included task shifting of some patient education and activation tasks [22] and collaboration in leadership and training implementation of diverse healthcare and social/community services sectors [19,20,21], consistent with promoting community resiliency [8]. This approach led to over 100,000 client service encounters for New Orleans residents [21]. Subsequently, this model, with adaptations for under-resourced communities of color, was evaluated outside of a post-disaster context in a program-level randomized trial in Los Angeles called Community Partners in Care (CPIC). CPIC compared a multi-sector coalition approach to individual program technical assistance to implement the expanded model of depression collaborative care across sectors. The coalition model was consistently associated with improved outcomes, ranging from mental health-related quality of life to reduced behavioral health hospitalizations and other outcomes, at 6-, 12- and 36-month follow-up [24,25,26,27]. A similar coalition approach was evaluated in a community-level randomized demonstration in Los Angeles of community resilience compared to standard disaster preparedness [28,29,30]. The collaboration across New Orleans and Los Angeles continued through the Patient Centered Outcomes Research Institute (PCORnet) and Community and Patient Powered Research Network (CPPRN), which supports new demonstrations, integrated datasets and a patient research registry [31,32].

In the months after the Great Flood of 2016, members of the CPPRN partnered with Louisiana-based disaster recovery experts, researchers, clinicians and community agencies to develop and implement a community resilience-oriented disaster recovery program, Resilient Baton Rouge (RBR), to build local capacity to promote mental wellness and reduce the burden of depression and its consequences following the floods. RBR was funded by the Robert Wood Johnson Foundation (RWJF), with fiscal sponsorship by the Baton Rouge Area Foundation, a major local philanthropic organization. Building on principles of community partnered participatory research [33] and the 13-year history of collaboration in addressing depression across Louisiana and Los Angeles, the goals of RBR were to use evidence-based models to boost the local ability of Baton Rouge to address common post-disaster behavioral health issues, using depression in adults as the first focus, and to create connections locally and nationally that would support community resilience and recovery. RBR had a focus on building collaboration among agencies, communities, government and other stakeholders, to bolster resilience in ways that are meaningful to communities [34,35] and strengthen long-term ability to adapt to natural disasters and other hazards [6]. We also sought to learn from the community what gaps in addressing mental health existed and what the strengths and priorities were for improvements to inform how the implementation occurred in this first phase and for planning for future recovery activities. In this case study report, we outline our project development and implementation strategies, initial results from first provider trainings and lessons learned that may be informative for other communities preparing for and responding to disasters.

## 2. Methods

### 2.1. Program Development

The narrative description of programs and processes implemented through RBR are drawn from descriptions of program goals and activities, meeting agendas and minutes, notes taken during training sessions and input from the authors and key program participants during project activities.

The overall approach of RBR was to build capacity to prepare for and respond to disaster-related behavioral health needs, as well as foster new collaborations to bolster community resilience. BRB had four main aims: (1) expand the local mental health infrastructure by providing support to hire new staff to provide direct services and training new and existing providers on evidence-based models of care, with a focus on collaborative care for depression and its adaptations for post-Katrina recovery and Community Partners in Care [18,19,20,21,22]; (2) plan and coordinate service delivery; (3) support and develop partnerships to promote community resilience-building activities; and (4) create a national community resilience learning collaborative through which experts could share resources and best practices, as well as provide input to the development of the local RBR program. 

Leadership structure: To initiate RBR, a leadership structure was developed that included: Program Director and Program Manager; liaisons to the New Orleans post-Katrina work and CPPRN and to the Los Angeles CPIC and CPPRN structure; and community and policy stakeholder advisory board, with co-chairs; and other stakeholder and academic consultants, as well as funder liaisons.

Program activities: To achieve our first aim, the RBR leadership team developed a competitive, sub-grant application process for agencies seeking support to expand behavioral health and other related service providers. With input of a community advisory board, RBR developed scoring criteria to evaluate applications. These included demonstrated need for additional resources in behavioral health, a feasible plan for implementation, commitment to collaboration in community resilience activities, service areas for populations affected by the Great Flood of 2016 and potential for impact and the sustainability of new services. Four members of the leadership team independently reviewed and scored the applications. Scores were aggregated to determine which agencies had been scored most highly, overall.

To address the second aim, we collaborated across Louisiana and Los Angeles partners to develop a training portfolio to support behavioral health practitioners, primary care providers, CHWs, other healthcare and social services providers in implementing evidence-based models of depression collaborative care, including support for medication management and clinical assessment, cognitive behavioral therapy (CBT), care/case management and health worker outreach, team-based and system support and component parts such as depression screeners and outcomes’ tracking, as well as patient and provider educational resources. As in our previous work, our toolkit included resources to support a wide range of community service organization staff, faith-based leaders, CHWs, volunteers and others in non-clinical roles to conduct outreach, offer education and make referrals for behavioral health services. One particular resource was manualized support for a psychoeducation program based on CBT, B-RICH, which recently has been the focus of a separate randomized trial, to facilitate awareness of CBT principles and methods for diverse populations through group sessions conducted by lay persons with ongoing supervision. To consolidate resources for RBR, a website was developed to include manuals, forms and links to websites relevant to the local community.

To address Aim 3, we created an advisory council comprised of stakeholders from state and local government, a representative of the major hospitals, mental health providers and community leaders involved in behavioral health in greater Baton Rouge. They advised on strategies for project implementation, promoting resilience and encouraging collaboration and engagement of flood-impacted communities. The advisory board met regularly and reviewed all major aspects of the project. Members facilitated linkages to other organizations and coalitions, as well as served as major partners for training events.

To address Aim 4, with support from the advisory board, RBR leadership focused on developing partnerships with grassroots organizations and governmental agencies. We identified experts throughout the country, particularly partners from around the Gulf region after the four 2017 hurricanes, to explore developing a National Resilience Learning Collaborative (NRLC) dedicated to sharing best practices for promoting disaster preparedness and community resilience and recovery in relation to behavioral health. This included, for example, participating on advisory boards or stakeholder boards of other initiatives and, based on those relationships, inviting stakeholders to preliminary meetings to discuss a learning collaboration for RBR.

### 2.2. Data Collection and Analysis

Qualitative/historical data: We collected meeting minutes during project council meetings and trainings. Notes were de-identified prior to analysis. Participants gave verbal consent to have notes collected and had the option to offer comments “off the record” if they did not wish to have their input recorded. However, it is important to note “off the record” comments would not have likely been analyzed in our results. To analyze qualitative data, we reviewed meeting minute and agendas. We identified several major themes that emerged. Two researchers then independently reviewed all documents and extracted examples to illustrate themes.

Training participant survey data: To initiate the process of describing context for training and potentially to track outcomes of training, we initiated a standard “new participant” survey, beginning with the first RBR depression collaboration care orientation and training session. We offered an optional 34-item survey to assess baseline capacity to address behavioral health among the health and social service providers participating in this group training. Study measures included current level of provision of services in a clinical or community setting, perceived depression knowledge and stigma, skill in providing services for individuals with depression, current case and care management techniques, current level of community outreach, inter-agency collaboration, readiness to implement quality improvement trainings, perceived barriers to providing optimal services, as well as demographics. Items were adapted from prior sources, including the CPIC administrator and provider surveys and our New Orleans post-Katrina work [18,21,36].

To analyze survey responses, we calculated univariate descriptive statistics, as well as bivariate analyses to assess differences between licensed health staff and non-licensed service providers, as well as differences between providers at health clinics and other community-based agencies. These comparisons were included to inform training goals for these subgroups of participants.

All research procedures were approved by the UCLA Institutional Review Board, which is also the coordinating IRB for the CPPRN project supporting the evaluation.

## 3. Results

Activities related to each of our four aims are described below and summarized in Table 1.

Aim 1: Among a total of nine organizations that applied for the competitive sub-grant process, we selected a total of four agencies to receive funds. All were federally-qualified health centers (FQHCs). Subgrants totaled approximately forty percent of the entire project budget. Agencies used funds to hire additional behavioral health staff including licensed clinical social workers, psychiatric nurse practitioners and CHWs. All subgrantees tracked progress on the number of new and existing flood-affected clients, as well as type of services delivered and submitted quarterly progress reports.

Aim 2: The first main orientation and training session was a two-day conference at a local community center approximately 10 months after the Great Flood. A total of 75 participants from a variety of local health services agencies, including the four subgrantee FQHCs, attended, as did staff from other community-based organizations. Participants included primary care providers, psychiatrists, outreach workers, case managers, care managers, counselors, social workers, community organizers, faith-based leaders, nonprofit agency staff and social services providers. All participants received training on the collaborative care model. We also offered break-out sessions with training tailored to various professional interests including strategies for community outreach for CHWs and case managers and CBT for therapists. The workshop also included structured networking activities and a session dedicated to assessing current community behavioral health capacity and needs.

Among the participants, 43 chose to respond to the optional provider survey. Respondents were primarily women (88%), and roughly half (51%) were African American. All reported having at least a college education. Three fifths identified as social workers or case managers, and just under two thirds (63%) reported working at a healthcare or mental health agency. Respondent demographics are summarized in Table 2.

Approximately half (49%) reported a moderate level of depression knowledge, and nearly three quarters (74%) believed they could improve on identifying, screening, assessing, educating and referring people for services. There were no statistically-significant differences on any survey items between people who worked at healthcare service agencies and those who worked in non-clinical settings. We found statistically-significant differences in depression knowledge, skills, care techniques, care management, use of resources and number of hours spent on clinical services provision between licensed and non-licensed providers. A summary of our bivariate analysis is included in Table 3.

As a follow up to requests made at the large group training, the RBR leadership team visited behavioral health and administration teams at three local hospital systems, three subgrantees clinics and one faith-based organization in the 3 months following the kick-off conference. We provided technical support for implementing components of the collaborative care model and assessed desire for follow-up trainings tailored to each agency. We also identified a total of 20 licensed therapists already practicing in the community who sought to advance their skills in delivering and training others on how to implement CBT. One of our team members with expertise in CBT conducted a “train the trainers” workshop for this group. Participants then had the option to participate in a 12-week program of ongoing technical assistance for implementation.

Based on community input, we offered additional training focused on implementing community outreach. For example, one of our subgrantee agencies invested its financial support in hiring CHWs to serve as a bridge for patients between primary and behavioral health. We trained a total of 11 administrators and other staff members in skills and processes to help implement this new role at the multi-site agency. We also offered a one-day, large group training session about one year into the project that focused on how CHWs, case managers and other non-clinical personnel can address depression through outreach, education, screening and referrals. Fifty people attended.

For Aim 3, we successfully hosted a total of five bimonthly advisory council meetings that included a total of 38 members. We also convened a total of nine monthly meetings with the four subgrantee organizations to increase coordination amongst the organizations and build collaborative solutions to shared issues. We also developed relationships with several local governmental agencies to begin a Capacity Building Activity Council, which met a total of four times. The group is now collaborating with the Mayor’s Office of Homeland Security and Emergency Preparedness (MOHSEP) and the region’s human services district to develop a replicable model for disaster recovery based on the Federal Emergency Management Agency’s (FEMA) National Disaster Recovery Framework (NDRF) [37]. We also collaborated with a community-based organization focused on homelessness and associated social determinants of health [38] to co-host a series of three Community Conversations, which were open dialogues with community members around issues of housing, health and disaster recovery. These Community Conversations are ongoing. We anticipate analyzing data collected at these events to inform future endeavors, such as the NRLC. We also hosted a discussion with a local faith-based leader about options for including faith-based institutions in collaborative care.

For Aim 4, our activities for exploring the NRLC included convening planning meetings, participation in the advisory process for another RWJF grantee developing web resources on community resilience for preparedness and participating in planning calls for a potential collaboration across areas affected by the 2017 Gulf hurricanes, followed by RBR leaders providing technical assistance to colleagues in the U.S. Virgin Islands and Houston for storm recovery. Follow-up activities discussed included hosting webinars and lessons learned from RBR and different projects across sites working on community disaster resilience and behavioral health recovery goals.

Thematic analysis of documents: We analyzed a total of 23 documents from project-related meetings and training events during the first year of the project. Participants in RBR activities identified several community strengths, including local community service organizations, non-profits and individuals who volunteered to help one another during and after the flood. Several social determinants of health were considered to be important in addressing behavioral health recovery. These included ongoing challenges with home rebuilding, insurance claims and homelessness. Community violence and its associated grief and trauma were mentioned at multiple meetings. Transportation was reported to be a factor in patients/clients missing appointments. Participants pointed out that health and social services were not equally distributed throughout the community, with wealthier areas with predominantly non-Hispanic white populations, having greater access to resources. Another factor participants believed worthy of consideration was the community’s high rate of HIV. In addition, advisory board members and training participants noted that efforts to address stigma associated with mental health would be vital to ensure that residents would seek services. The need for a reliable up-to-date resource guide was mentioned several times. Behavioral health staff and administrators also recognized the importance of self-care for providers in order to avoid burnout and secondary trauma. In terms of desire for training and capacity building, RBR participants perceived a need for support to conduct outreach and define a role for faith community leaders. Child trauma was a common theme, with participants mentioning a need to support schools in addressing the issue and requesting resources to give parents. The results are summarized in Table 4.

## 4. Discussion

In this case study, we describe the development of a community-academic partnered approach to building capacity to address behavioral health and promote community resilience in a disaster-affected city. RBR is an implementation project with a descriptive evaluation, largely for quality improvement and program development purposes, rather than a formal research study. The initiative coupled the provision of grants to services agencies to build capacity for behavioral health services with shaping the services offered through these contracts, as well as more broadly in the community, through orientation and training in an expanded version of depression collaborative care for healthcare and community settings, with a particular emphasis on case management, outreach and CBT. This emphasis was because system stakeholders identified an initial priority for psychosocial services support as complementary to more medically-oriented services such as clinical assessment and medication management. 

We found that it was feasible to support system partners in building services capacity and that it was acceptable to do so following an evidence-based model. The local history of applying the collaborative care model with regional and more distant geographic support may have facilitated that acceptability and engagement. Although we were generally able to execute the project as planned, we found that implementation was somewhat delayed initially by the need to create new project management documents and protocols. Future work might benefit from the development of standardized project operations manuals for subcontracts and planning for ramping up of staffing capacity immediately post-disaster to avoid delays in project execution, even when training protocols exist. Maintaining the availability of training protocols may also be key to rapid start-up to support recovery.

With regard to training, we were able to adapt and use existing materials on collaborative care, including CBT and community outreach. Local demand for tools to address behavioral health was strong, as evidenced by the number of training participants being almost double our initial estimates at several events. We found, not surprisingly, that licensed and non-licensed providers differed significantly in their capacity to address depression. This confirmed that having some specialized educational tracks based on occupation was appropriate. While those who were licensed had opportunities to train, we also found there was great value with the trainings among the non-licensed participants, as many expressed a need for more opportunities to learn and support their work. The lack of significant differences between providers at healthcare organizations and social services agencies suggests that it was appropriate to train members of such agencies simultaneously, and doing so provided what was perceived to be a valuable networking opportunity. This experience also parallels lessons learned from CPIC and work in post-Katrina New Orleans [18,21,36]. The feasibility of implementing trainings and adapting to local needs and assets was enhanced by the partnership history of collaboration in adapting collaborative care to community-wide implementation including in a disaster context. This may be an important set of resources and experience to share particularly through the evolving NRLC. Although we focused on adult depression, similar models and experience would also be helpful in other areas including for children and adolescents, as well as PTSD and other behavioral health conditions. This gap emerged in some of our meetings with interested stakeholders. We received anecdotal feedback from participants and community residents. Training participants asked for more tools and cited instances of clients speaking about reduced stigma around seeking help for depression and PTSD. We are actively exploring options to build on other resources in these areas [39] for future training sessions and plans for a second phase of RBR.

Although RBR primarily centered on capacity building for behavioral health, stakeholders also commented that our role as a convener was also valuable for fostering connections that may contribute to recovery and resilience more generally. For example, our subgrantee meetings were initially implemented to ensure consistent communication between project staff and the agencies and to develop standardized reporting protocols. However, our participants found them most valuable for facilitating communication across agencies and sharing best practices. Similarly, members of our advisory council provided invaluable suggestions for project execution, but the act of simply bringing together behavioral health experts and council members with other areas of expertise also served the role of forging important connections. In seeking to collaborate with existing disaster recovery efforts to avoid duplication of services, existing relationships of RBR leaders or advisory board members with grassroots organizations already embedded in and trusted by the community lent credibility to our efforts. For example, Assisi House, Inc., a small 501c3 organization that conducts research and programming around housing and public health, was already working on an RWJF project before the flood and had worked with local universities and the local community around housing outcomes [39]. Their methodology and outreach efforts were directly impacted by the Great Flood. Partnership with them allowed RBR leaders to engage in small community conversations with local residents, enhancing the capacity to learn about community connections and facilitate entry into the community.

We were not surprised that training participants and advisory council members identified social determinants of health such as community violence and housing as significant issues. This work began shortly after a violent summer in which Baton Rouge police killed Alton Sterling, six police officers were shot and protests against police brutality made national news [40]. In addition, many area residents were just beginning to rebuild their homes after the Great Flood of 2016. Future resilience building and disaster recovery efforts must take into account existing and recurring community trauma, as well as social issues, and develop tailored services to address these concerns. Because of such concerns, there was broad agreement among stakeholders on the need for intervention in behavioral health, as well as in building collaborations to more broadly address resilience and wellness as a community.

During the first year of this project, an unprecedented four major hurricanes hit the United States, highlighting the need for coordinated disaster response. We are hopeful that our NRLC will continue to develop and expand, enhancing opportunities for communities recovering from and preparing for disasters to learn from one another and share community engagement and intervention strategies. Similarly, we are hopeful that our participation in implementing the NDRF in Louisiana will help ensure that behavioral health is adequately addressed in disaster planning and recovery.

This case study is limited in that it describes the experiences of only one community in response to one major disaster. The number of respondents to the baseline provider survey was relatively small (N = 43), and results may be biased if the 32 providers who attended our initial training, but did not respond, are somehow different than those who did. We did not have notes for all meetings and training sessions. Nonetheless, this paper provides insight into how one community built on existing models of capacity building for behavioral health in a post-disaster setting, to support an operational model (services contracts, capacity trainings, partnership development, national collaborative) that may be replicable in other communities seeking to enhance resilience and behavioral health.

Future directions for RBR may include continuing to provide technical assistance and capacity building, further collaborations with faith-based organizations to bolster community outreach and cultural sustainability and addressing issues related to child and adolescent trauma, as well as other behavioral health conditions (e.g., substance misuse) that may be exacerbated post-disaster.

## Figures and Tables

**Table 1:** Resilient Baton Rouge Year 1 activities.

Activity	# of Events	Total # of Participants (mean per event)
Aim 1: Build mental health services delivery capacity		
Large group training for all provider types	1	75
Training for community health workers/case managers	1	50
Training for subgrantee agency	1	15
CBT Train the Trainer	1	23
CBT Train the Trainer Technical Assistance Sessions	2	4 (2)
Aim 2: Plan and coordinate services		
Advisory council meetings	4	38 (15)
Subgrantee meetings	9	42 (5)
Aim 3: Develop partnerships to promote community resilience		
Community Conversations	3	10
Capacity Building Working Group	4	18 (5)
Aim 4: Develop the National Resilience Learning Collaborative (NRLC)		
Conference calls with colleagues that experienced disasters in 2017	3	25

**Table 2:** Demographics of baseline provider survey respondents.

Demographics, N = 43	N	%
Age	49.2 ± 11.4	
Female	38	88
Racial or ethnic background (*n* = 41)		
▫ Black/African-American	21	51
▫ White	18	44
▫ Other	2	5
Education (*n* = 42)		
▫ College	12	29
▫ Masters or above	30	71
Occupation		
▫ Social Worker/Case Manager	26	60
▫ Nurse	6	14
▫ Other	11	26
▫ Licensed	33	77
▫ Non-licensed	10	23
Agency (*n* = 41)		
▫ Health/Mental Health Center	26	63
▫ Other	15	37

**Table 3:** Baseline provider need for training to address depression.

Measures, Standardized Cronbach’s α for Scales with Two or More Items	Total N = 43 M ± SD	NL M ± SD	Licensed M ± SD	Statistic *t* (df)	*p*	Total N = 41 M ± SD	Non-HC M ± SD	Healthcare M ± SD	Statistic *t* (df)	*p*
**Depression knowledge**, α = 0.21 (3 items, 1 = strongly agree, 5 = strongly disagree) ^a^	1.5 ± 0.5	1.9 ± 0.3	1.4 ± 0.5	t_(37)_ = −2.8	0.008	1.5 ± 0.5	1.5 ± 0.4	1.6 ± 0.6	t_(35)_ = 0.2	0.817
**Depression skill**, α = 0.89 (7 items, 1 = not at all skilled, 4 = very skilled) ^b^	2.8 ± 0.9	1.8 ± 0.7	3.1 ± 0.6	t_(39)_ = 5.7	<.001	2.8 ± 0.9	2.7 ± 1.0	2.8 ± 0.8	t_(37)_ = 0.3	0.757
**Depression stigma**, α = 0.56 (3 items, 1 = strongly agree, 5 = disagree) ^c^	3.8 ± 0.7	3.6 ± 0.7	3.9 ± 0.7	t_(41)_ = 1.4	0.175	3.8 ± 0.7	3.9 ± 0.8	3.8 ± 0.7	t_(39)_ = −0.1	0.885
**Perception of systemic barriers**, α = 0.60 (3 items, 1 = doesn’t limit, 3 = limits a great deal) ^d^	1.5 ± 0.5	1.7 ± 0.7	1.5 ± 0.4	t_(33)_ = −0.9	0.352	1.5 ± 0.5	1.6 ± 0.6	1.4 ± 0.4	t_(31)_ = −1	0.333
**Depression care techniques**, α = 0.95 (9 items, 1 = never, 5 = always) ^e^	2.6 ± 1.0	1.9 ± 1.1	2.8 ± 0.9	t_(34)_ = 2.4	0.023	2.7 ± 1.0	2.3 ± 1.0	2.9 ± 0.9	t_(32)_ = 1.7	0.091
**Depression care management**, α = 0.92 (5 items, 1 = never, 5 = always) ^f^	2.6 ± 0.9	1.7 ± 0.8	2.8 ± 0.8	t_(35)_ = 3.4	0.002	2.6 ± 0.9	2.3 ± 1.0	2.8 ± 0.8	t_(33)_ = 1.5	0.148
**Resource Use in past 6 months**, α = 0.59 (4 items, 1 = didn’t use, 3 = used a lot) ^g^	1.7 ± 0.5	1.3 ± 0.3	1.8 ± 0.5	t_(36)_ = 2.3	0.025	1.7 ± 0.5	1.5 ± 0.3	1.8 ± 0.6	t_(34)_ = 1.6	0.113
**Clinical services provision, hours per week** (0 = 0 hours, 5 = more than 40 hours) ^h^	1.5 ± 1.7	0.1 ± 0.3	1.9 ± 1.7	t_(38)_ = 3	0.005	1.5 ± 1.7	0.7 ± 1.4	1.9 ± 1.7	t_(36)_ = 2.2	0.036
**Community services provision, hours per week** (0 = 0 hours, 5 =more than 40 hours) ^h^	1.2 ± 1.6	1.3 ± 1.4	1.2 ± 1.6	t_(36)_ = −0.1	0.884	1.2 ± 1.6	1.4 ± 1.6	1.1 ± 1.6	t_(34)_ = −0.4	0.687
**Community outreach** (1 = 0%, 5 = 75–100 % of time) ^i^	1.9 ± 1.1	1.6 ± 1.0	1.9 ± 1.2	t_(40)_ = 0.8	0.42	1.8 ± 1.1	2.1 ± 1.1	1.7 ± 1.1	t_(38)_ = −1.1	0.291
**Improving organization services**, α = 0.97 (4 items, 1 = not import, 7 = extremely import) ^j^	6.0 ± 1.5	5.6 ± 1.9	6.2 ± 1.4	t_(39)_ = 1.0	0.345	6.2 ± 1.3	5.6 ± 1.7	6.5 ± 0.9	t_(37)_ = 1.9	0.06
**Improving client services**, α = 0.94 (3 items, 1 = not import, 7 = extremely import) ^j^	6.2 ± 1.2	6.1 ± 1.1	6.3 ± 1.3	t_(38)_ = 0.4	0.718	6.4 ± 0.9	6.1 ± 0.9	6.5 ± 0.8	t_(36)_ = 1.4	0.157

^a^ Possible scores range from 1 to 5, with lower scores indicating greater depression knowledge [36]; ^b^ Possible scores range from 1 to 4, with higher scores indicating greater perception of skills [36]; ^c^ Possible scores range from 1 to 5, with higher scores indicating less depression stigma [36]; ^d^ Possible scores range from 1 to 3, with lower scores indicating less barriers; ^e^ Possible scores range from 1 to 5, with higher scores indicating greater use of depression care techniques [36]; ^f^ Possible scores range from 1 to 5, with higher scores indicating greater use of depression case management tasks [36]; ^g^ Possible scores range from 1 to 3, with higher scores indicating more frequent use of the resources for individuals who showed symptoms of depression and came to this organization for services; ^h^ Possible scores range from 0 to 5, with higher scores indicating more hours providing services [36]; ^i^ Possible scores range from 1 to 5, with higher scores indicating greater percentage of working time spent on engaging in community outreach for depression; ^j^ Possible scores range from 1 to 7, with higher scores indicating more improtant for improving services.

**Table 4:** Summary of themes from Year 1 Resilient Baton Rouge project activities.

Theme	Examples
Community strengths	
Informal and formal networks	Cajun Navy and neighbors helping one another during and after flood Non-profit organizations, faith communities
Social determinants of health/community issues	
Housing	Residents face ongoing issues with rebuilding homes, insurance claims and lack flood insurance Homelessness is a major concern
Community violence	Gun violence is an ongoing issue Trauma and grief related to violence should be considered when addressing behavioral health
Transportation	Patients/clients miss appointments due to a lack of transportation
Health Access inequities	Some areas of the community have better access to health and social services Differences tied to geography and race
HIV	Local community has one of the country’s highest HIV rates
Stigma	Community residents may be hesitant to discuss behavioral health
Behavioral health staff needs	
Resource guide/networking opportunities	Community resources change rapidly There are existing resources guides, but they need to be streamlined/updated There is a desire for networking between agencies
Self-care	Staff may experience secondary trauma from assisting clients Administrators and staff recognize a need for staff support for self-care
Desire for training/capacity building	
Training for non-clinical providers	Need for training to help address stigma, promote outreach and address cultural competence Interest in role of faith communities/religious leaders in behavioral health promotion
Child trauma	Need to address trauma in schools Clinical and non-clinical staff need trauma resources to share with parents
Support for healthcare agencies	Desire for technical assistance with data tracking and coordination of services Interest in help for specific populations (e.g., pregnant women)
